# The effect of COVID-19 on completed suicide rate in Iran: an Interrupted Time Series study (ITS)

**DOI:** 10.3389/fpubh.2025.1387213

**Published:** 2025-02-13

**Authors:** Azadeh Nouhi Siahroudi, Seyed Saeed Hashemi Nazari, Mahshid Namdari, Mohammad Hossein Panahi, Seyed Amirhosein Mahdavi, Ali Khademi

**Affiliations:** ^1^Department of Epidemiology, School of Public Health and Safety, Shahid Beheshti University of Medical Sciences, Tehran, Iran; ^2^Safety Promotion and Injury Prevention Research Center, Shahid Beheshti University of Medical Sciences, Tehran, Iran; ^3^Legal Medicine Research Center, Legal Medicine Organization of IRAN, Tehran, Iran; ^4^Legal Medicine Research Center, Iranian Legal Medicine Organization, Tehran, Iran

**Keywords:** complete suicide, COVID-19 disease, Interrupted Time Series method, Iran, Rate incidence

## Abstract

**Background:**

Suicide represents a critical public health concern and one of the most devastating forms of death. Based on a report from the World Health Organization, around 700,000 deaths by suicide occur globally each year. In 2019, the worldwide suicide mortality rate was 9.0 per 100,000 people, while in Iran, this rate has been reported to be an average of 5.2 per 100,000. Suicide is influenced by various factors spanning individual, relational, community, and social domains, all of which may elevate the risk of suicide and related death. One significant factor potentially impacting this issue was the COVID-19 pandemic, which may have affected these trends by disrupting individuals’ social interactions and gatherings. To explore this further, the present study was carried out to investigate the impact of the COVID-19 pandemic on the changes in suicide rates leading to death in Iran.

**Methods:**

This study was designed using an Interrupted Time Series approach combined with negative binomial regression. Seasonal variations were adjusted for using the harmonic method. The research sample comprised 63,514 suicide-related deaths recorded between April 20, 2009, and March 20, 2023. Suicide mortality data were sourced from the National Legal Medicine Organization, while population statistics were obtained from the official website of the Statistical Center of Iran. The study analyzed trends in suicide incidence both prior to and during the COVID-19 pandemic. The period used to evaluate pandemic-related changes in Iran began in May 2020, following the World Health Organization’s declaration of COVID-19 as a global public health emergency. Descriptive analyses were performed using Stata software, and trend assessments through the Interrupted Time Series (ITS) method were conducted using R software and the “lmtest” statistical package.

**Results:**

The changes in the incidence of suicide during the study increased by 1.003 monthly (*p* < 0.001). This rate increased by 1.1 (*p* < 0.001) compared to the times before the onset of the pandemic after entering the effect of the COVID-19 pandemic in the model. When the interaction effect of time with the COVID-19 pandemic was added to the base model, no significant relationship was observed.

**Conclusion:**

Before the COVID-19 pandemic, suicides in Iran had a proportional increasing trend. However, three months after the pandemic, an increasing trend in the level of suicide deaths was observed. Most likely, the COVID-19 pandemic phenomenon had an impact on the occurrence of suicide.

## Introduction

Suicide has been a significant public health concern impacting individuals of all ages, genders, and regions (1–3). According to the latest report of the World Health Organization, more than 700,000 people lose their lives due to suicide every year (4). The age standardized suicide rate in the world was 9.0 per 100,000 population in 2019, which varied between countries from less than two to more than 80 per 100,000 population. In terms of age, suicide is the fourth leading cause of death among young people aged 15 to 29 years in both sexes (5, 6). Every death due to suicide is a tragedy (7) therefore, paying attention to the causes of suicide and examining its impact from other factors is very important and necessary. Stressful life events, as one of the main risk factors for suicide, are increasing as a global outbreak. One of these stressful events in late 2019 was the outbreak of the new coronavirus (COVID-19), which caused significant stress and social turmoil at the national and global levels, in addition to the casualties caused by the disease (8). In December 2019 and according to the identification of the first case of COVID-19 in Wuhan, China, the World Health Organization announced on January 31, 2020 that the outbreak of this disease was a public health emergency of international concern (9, 10) and took actions such as restricting travel, closing borders, limiting international relations, quarantining, losing jobs, changing lifestyle, increasing deaths and social isolation of the population along with fear and horror of the disease, causing mental problems such as stress, anxiety and depression to increase along with this global pandemic (11, 12). Therefore, COVID-19 is not only an infectious disease with relatively high transmission and mortality (13) but is also considered a serious threat to mental health because social distancing, which was implemented as one of the necessary initial measures to reduce the speed of virus transmission and protect people from COVID-19, could have detrimental secondary effects on unemployment, loneliness and previous mental illnesses of individuals, all of which are known as risk factors for suicide (14). It seems that the side effects of actions that were imposed and implemented by governments to prevent the transmission of new cases of infection caused by COVID-19 (such as social distancing and preventing gatherings) have caused changes in the direction of decreasing or increasing suicide cases (15, 16). According to the comparable estimates of the World Health Organization in 2019, the death rate from suicide in Iranian men and women was 5.2 per 100,000 population, so that this rate was calculated as 4.5 in men and 2.7 per 100,000 population in women (17–19). According to studies that have been performed in Iran and other countries of the world since the beginning of the COVID-19 pandemic, mental illnesses, especially depression, anxiety, and stress, have increased in all groups and occupations (20–25). The primary aim of this study was to examine whether the rise in COVID-19 cases has resulted in an increase or decrease in completed suicides in Iran. To achieve this objective, this objective, the study analyzed the trend of completed suicides across two periods: before and after the World Health Organization declared COVID-19 a mental health emergency impacting human societies. Interrupted Time Series analysis was used to assess its potential effect on suicide statistics within Iran.

## Methods

This research was carried out using the framework of ecological studies. The information for the study was obtained and gathered from two main sources:

Records of suicide cases that resulted in death between 2009 and 2023, registered with a suicide diagnosis as the determined cause of death by Iran’s Legal Medicine Organization. 2- Essential demographic details and indicators retrieved from the official portal of the Statistical Center of Iran.

After obtaining the study ethics code from the Research Ethics Committee of School of Public Health and Neuroscience Research Center - Shahid Beheshti University of Medical Sciences and presenting it to the Legal Medicine Organization of Iran, the research team received an SPSS file with annual demographic data of individuals who had died by suicide during the study period (2009–2023). The data underwent initial checks for completeness, duplicate removal, and cleaning. Suicides resulting in death were then compiled monthly and annually using Stata software, with categorization based on study variables. Descriptive statistics, incidence rates, and suicide trends were subsequently calculated and analyzed.

Analytical evaluations were performed using the Interrupted Time Series approach, applying segmented regression techniques within R software. This method was chosen because the Interrupted Time Series design is among the most robust quasi-experimental approaches for assessing the effectiveness of health interventions or the impacts of unplanned events and natural occurrence (26), such as the COVID-19 pandemic and etc., which likely influenced community health.

To calculate the age-standardized suicide rate over the 14-year study, the Direct Standardization Method was utilized, based on the World Standard Population framework provided by the World Health Organization for use by countries.

An Excel table containing aggregated data on completed suicides is needed for input into the R software to execute discrete time series commands. This table should consist of seven distinct columns, each described as follows:
The first column represents the year in which suicides resulting in death occurred (Year).
The second column indicates the month of these suicides (Month). Since the study spans 14 years, with each year containing 12 months, this creates 168 rows in which the year is repeated across 14 consecutive years, and the month ranges from 1 to 12, repeated across the 12 months of each year.
The third column is assigned to the outcome variable, which reflects the number of suicides (1). These figures were obtained from the cleaned SPSS dataset and recorded in the Excel spreadsheet, corresponding to the specific month and year of the suicides under examination.
The fourth column encompasses the time variable (Time), used to track changes in the outcome variable over the study period. This variable is numerically coded, starting at 1 for statistics corresponding to April 20, 2009. It progresses sequentially, culminating at 168, denoting March 20, 2023.
The fifth column is designated to the variable “level” in order to analyze changes or interventions, as represented by the term (COVID) in the table. This variable helps to measure the presence or absence of the event under observation by comparing changes in the outcome variable against the counterfactual level. The counterfactual level represents a scenario in which the trend of suicide data is predicted, calculated, and illustrated as though the event under observation—in this case, the COVID-19 pandemic—had not occurred.

In this explanation, the initial data in the “level” column (COVID) corresponds to the period from April 20, 2009, to early May 2020, when the World Health Organization classified the COVID-19 pandemic as a public health emergency affecting human societies (27). During this time frame, the code “zero” represents the absence of the event in question. From that point onward until the conclusion of the study period (March 20, 2023), the code “one” signifies the presence of the event under investigation. Therefore, the first 133 entries have been assigned the code “zero,” indicating the event’s absence, while the subsequent 35 entries are marked with the code “one,” reflecting the event’s occurrence.

Column six, the country’s population during the years 2009 April-2023 March is shown, which includes the census population or its estimate in the years between the census, which has been extracted from the official website of the Statistical Center of Iran.

The last column, the seventh column, expresses the standardized population of the country. In order to calculate the standard population of the country, the standard population table prepared by the World Health Organization for countries for the years 2005 to 2025 was used, and with the help of the Direct Standardization Method and estimation of the proportions related to each year, the Standardized Population was calculated for each year separately.

An example of the table prepared in the Excel file for recalling the study variables to run the Interrupted Time Series by the segmented regression method in R-Studio software is summarized in Table 1

**Table 1:** Sample data set for the study to be called in R-Studio software for discontinuous time series analysis

**Year**	**month**	**Suicide**	**time**	**covid**	*pop	*stdpop
20 April 2009	1	257	1	0	**73202**	**79039**
**...**	**...**	**...**	**...**	0	...	...
**2009**	**12**	**233**	**12**	0	**73202**	**79039**
**...**	**...**	**...**	**...**	0	**...**	**...**
**...**	**...**	**...**	**...**	0	**...**	**...**
**...**	**...**	**...**	**...**	0	**...**	**...**
2010	12	349	132	0	82710	83718
2020	1	376	133	0	83409	83512
2020	2	438	134	1	83409	83512
**...**	**...**	**...**	**...**	**...**	**...**	**...**
2020	12	457	144	1	83409	83512
**...**	**...**	**...**	**...**	**...**	**...**	**...**
**...**	**...**	**...**	**...**	**...**	**...**	**...**
20 March 2023	12	366	168	1	84700	85334

*Population and standardized population numbers are rounded to the nearest 1,000.

After preparing the data, it was necessary to pay attention to some points to use the time series method to implement the Interrupted Time Series Method. One of these points was to draw graphs with the help of which many characteristics of the study are detected, such as the presence or absence of trends, seasonal variations, frequencies and unusual (random) variations. By drawing diagrams, it is possible to investigate the autocorrelation and seasonal effect in the residuals of the model (26, 28), because the time variable can distort the results by creating a seasonal effect, so it is necessary to pay attention and adjust it (26).

In the first step, by drawing a distribution graph between the independent and dependent variables, the trend of data occurrence is investigated before the intervention. Because the trend of the data before the intervention indicates the trend of the variable studied over time. Based on the diagram drawn at this stage, it was found that the suicide data in this study have a linear trend with a seasonal effect.

In the second step, a statistical method was selected that showed a change in the effect of the intervention. Since the dependent variable (incidence) was calculated as the number of complete suicides and the nature of the data was discrete enumeration, and due to the over dispersion among the data, the negative binomial regression method was used. In the third step, to investigate the seasonal effect, the residuals of the model and the existence of partial autocorrelation and autocorrelation were drawn, and the examination of the graphs confirmed the seasonal trend in the data, and the harmonic method was used to adjust the seasonal effect. Due to the detailed presentation and review of the harmonic method, it is recommended that the respected readers refer to the following sources [1- Qiang Zhou et al.’s paper titled A novel regression method for harmonic analysis of time series and 2-James Lopez Bernal et al. (Interrupted Time Series regression for the evaluation of public health interventions: a tutorial)].

In the final step, the changes in the model’s slope were examined by analyzing the interaction between time and intervention. Ultimately, the segmented regression model used to evaluate shifts in the level and slope of the annual suicide incidence rate was formulated as follows. Given that the data may involve either a single group or comparisons across several groups, both single-group and multi-group methods can be applied. However, since the analysis in this study was conducted using a single group, the corresponding formula is as follows.

Yt=β0+β1T+β2Xt+β3TXt+εt

Yt is the indicator of the outcome variable at time t after the intervention or nonintervention.

Β0 is the baseline level of the outcome variable at time 0 = T (intercept).

Β1 is the indicator of the change in the outcome variable for one unit increase in time T (slope of the line before the intervention).

T is the indicator of the time elapsed from the beginning of the study with a unit that indicates the frequency of observations, such as month or year.

Β2 is the indicator of the change in the outcome level following the intervention.

Xt is the same binary variable that indicates the intervention or nonintervention of the outcome under study.

Β3 is the difference between the slope of the line before and after the intervention.

TXt is the interaction effect of time and intervention, which is also called the slope change after the intervention.

ε t is the model error or random error in time unit (t) (26, 29).

By fitting the Interrupted Time Series regression, the effect of time and intervention (COVID) with the multiplicative effect of time and intervention (interaction effect) were examined. ANOVA was used to compare the related models. The Interrupted Time Series analysis of the data was performed using R software at a significance level of 5 percent.

## Results

After extracting the demographic variables of people who died due to suicide from April 2009 to March 2023, it was found that 63,566 cases of suicides leading to death from all over Iran were recorded by the country’s forensic medicine, of which 52 cases of suicide were unknown and unrecoverable, which were excluded from the Interrupted Time Series analyses. In order to inform the readers of the article about all the missing cases in the studied variables, their number was included in the demographic and epidemiological tables for the transparency of the researchers’ work, but due to the fact that less than 10% of the missing cases in each variable were eliminated during the analysis. As a result, by excluding 52 cases unknown in terms of the month of suicide, the total number of suicides resulting in death in Iran during the 14 years from April 2009 to March 2023 was reported to be 63,514, of which 45,092 (70.9%) were male and 18,473 (29.1%) were female. The mean age of those who died by suicide was 33.9 years with 15.2 standard deviations. 77.6% of the patients who died by suicide were in the age group of 20–59 years, of which 45.5% were in the age group of 30–59 years. The lowest suicides occurred in the age groups of less than 10 years (0.2%) and over 60 years and older (7.4%), respectively. The suicide rate in men was 2.5 times that of women. However, this ratio has been very close to one in some provinces. In terms of suicide season, 34,406 (54.2%) deaths occurred in spring and summer and 29,108 (45.8%) in autumn and winter.

All the variables studied in this study, including marital status, occupation, education, province of residence of the deceased due to suicide, and the month in which the suicide occurred, were also investigated, and the results of demographic and epidemiological characteristics are summarized in Tables 2, 3, respectively.

**Table 2:** Demographic data of suicide deceased in Iran from April 20, 2009 to March 20, 2023.

Age indicators	Number	Percentage%
Mean	33.9	-
Median	30	-
Mod	20	-
Standard deviation	15.2	-
Age groups		
Less than 10 years	139	0.20%
10–19 years	9,445	14.90%
20–29 years	20,177	31.70%
30–59 years	28,906	45.50%
60 years and more	4,701	7.40%
Unknown	198	0.30%
Gender		
Male	45,092	70.90%
Female	18,473	29.10%
Unknown	1	0.00%
Marital status		
Single	28,408	44.70%
Married	31,607	49.70%
Divorced	2,115	3.30%
Widow	628	1.00%
Unknown	808	1.30%
Education level		
Illiterate, primitive, first high school	19,618	30.90%
Secondary High school, diploma and pre-university	36,438	57.30%
University education (postgraduate, bachelor’s degree and above)	6,316	9.90%
Unknown	1,194	1.90%
Job		
Housewife	13,734	21.60%
Worker	6,973	11.00%
Employee, doctor, teacher, judge, professor, psychologist, clergy man, soldier-military	4,446	7.10%
Unemployed	7,933	12.50%
Retired	2056	3.20%
Student, university student	6,337	10.00%
Driver, farmer	3,332	5.20%
Street vendor, smuggler, other	1,012	1.60%
Other self-employed occupations	16,854	26.50%
Unknown, child under 7 years old	889	1.40%

**Table 3:** Provincial divisions based on the zoning of the ministry of interior.

Region	Number	Percentage%
Region one: Tehran, Qazvin, Mazandaran, Semnan, Golestan, Alborz and Qom provinces	17,231	27.10%
Region two: Isfahan, Fars, Bushehr, Chaharmahal and Bakhtiari, Hormozgan and Kohgiluyeh and Boyer-Ahmad provinces	11,009	17.30%
Region three: East Azerbaijan, West Azerbaijan, Ardabil, Zanjan, Gilan and Kurdistan provinces.	12,811	20.20%
Region four: Kermanshah, Ilam, Lorestan, Hamedan, Markazi and Khuzestan provinces	15,315	24.10%
Region five: Razavi Khorasan, South Khorasan, North Khorasan, Kerman, Yazd, Sistan and Baluchestan provinces.	7,163	11.30%
Unknown region	37	0.06%

Season		
Spring	16,835	26.50%
Summer	17,571	27.60%
Autumn	14,460	22.70%
Winter	14,648	23.00%
Unknown	52	0.10%

**Figure 1:**
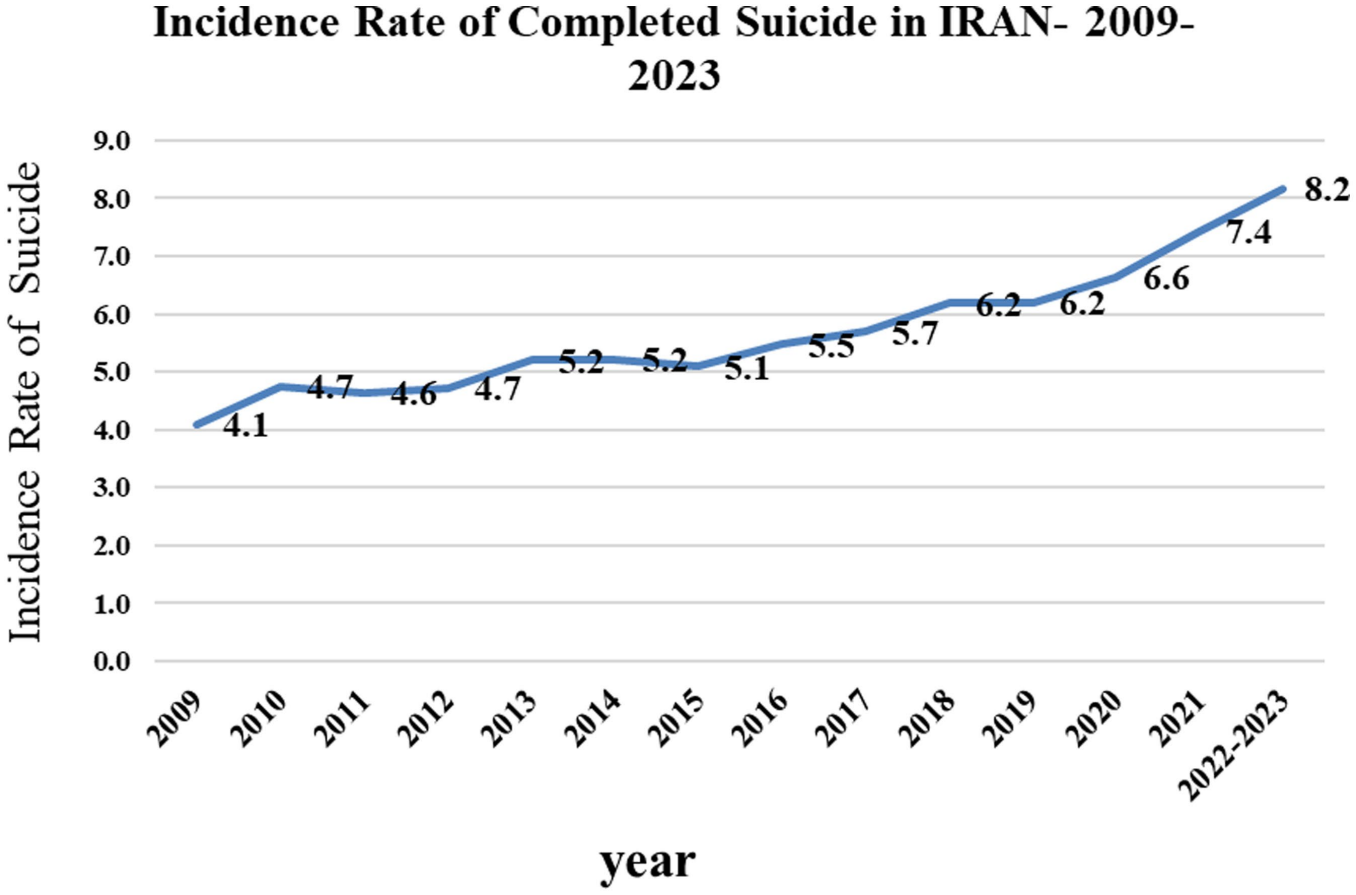
Trend of suicide rate leading to death from 2009 to 2011 in IRAN.

The trend of suicides from April 2009–March 2023 showed that the incidence of suicides increased from 4.1 in 2009 to 8.2 per 100,000 population in 2023. This upward trend can be seen in Figure 1.

**Figure 2:**
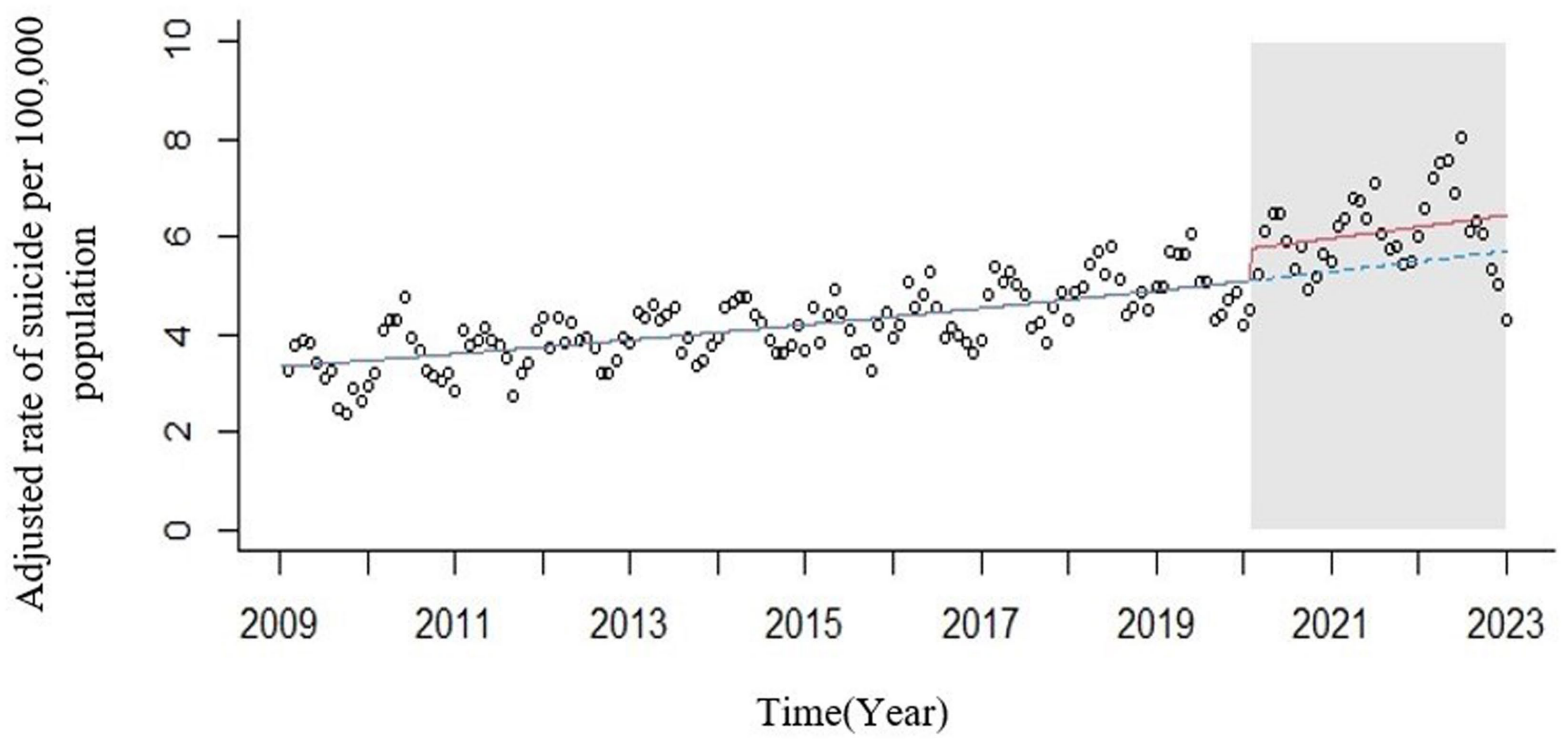
ITS plot of standardized incidence of suicide deaths in IRAN from March 2009 to 20,223 compared to the counterfactual level (circles = observed rates; red line = modeled rates fitted to the data; blue line = counterfactual level. Gray area = Announcement of mental health orientation following the COVID-19 pandemic by the WHO).

In the Interrupted Time Series analysis that was adjusted by negative binomial regression and using the harmonic method of season, it was determined that the incidence of suicide increased by 1.003 times per month, and this rate increased by 1.1 times in the presence of COVID-19 disease. The increase in the standardized incidence of suicide increased with increasing time and the presence of the COVID-19 pandemic, and this increase was significant (*p* < 0.001) (Figure 2).

In the study of the interaction effect of time and intervention on the incidence of suicide deaths, it was observed that the interaction effect of COVID and time does not cause a significant change in the graph and the incidence of suicides (*p* ≥ 0.05). The comparison of the Akaike criterion between the two models also confirmed the result of the ANOVA test, showing that each of the variables of time and COVID-19 had a positive and significant impact on the increase in standardized suicides, which caused an increase in the level of suicide compared to the time before the COVID-19 pandemic. However, the interaction effect between COVID-19 and time was not significant. The summarized results in Table 4 show this study.

**Table 4:** Comparison of implemented models to choose the final model of Interrupted Time Series.

Model name	Index title	Standardized incidence rate ratio exp (7)	Confidence interval 95% (Confidence interval)	Significance level (*p* value)	Akaike index (AIC)
Model 1 (Time and COVID-19)	Base value (Intercept)	0.003	0.003–0.003	0	1641.696
	Intervention (covid)	1.108	1.003–1.004	0	
	Time (time)	1.003	1.055–1.205	0	
Model 2 (Time and COVID-19 interaction)	Base value (Intercept)	0.003	0.003–0.003	0	1643.329
	Intervention (covid)	0.98	0.662–1.451	0.921	
	Time (time)	1.003	1.003–1.004	0	
	Time Intervention (covid* time)	1.001	0.998–1.003	0.54	

**Figure 3:**
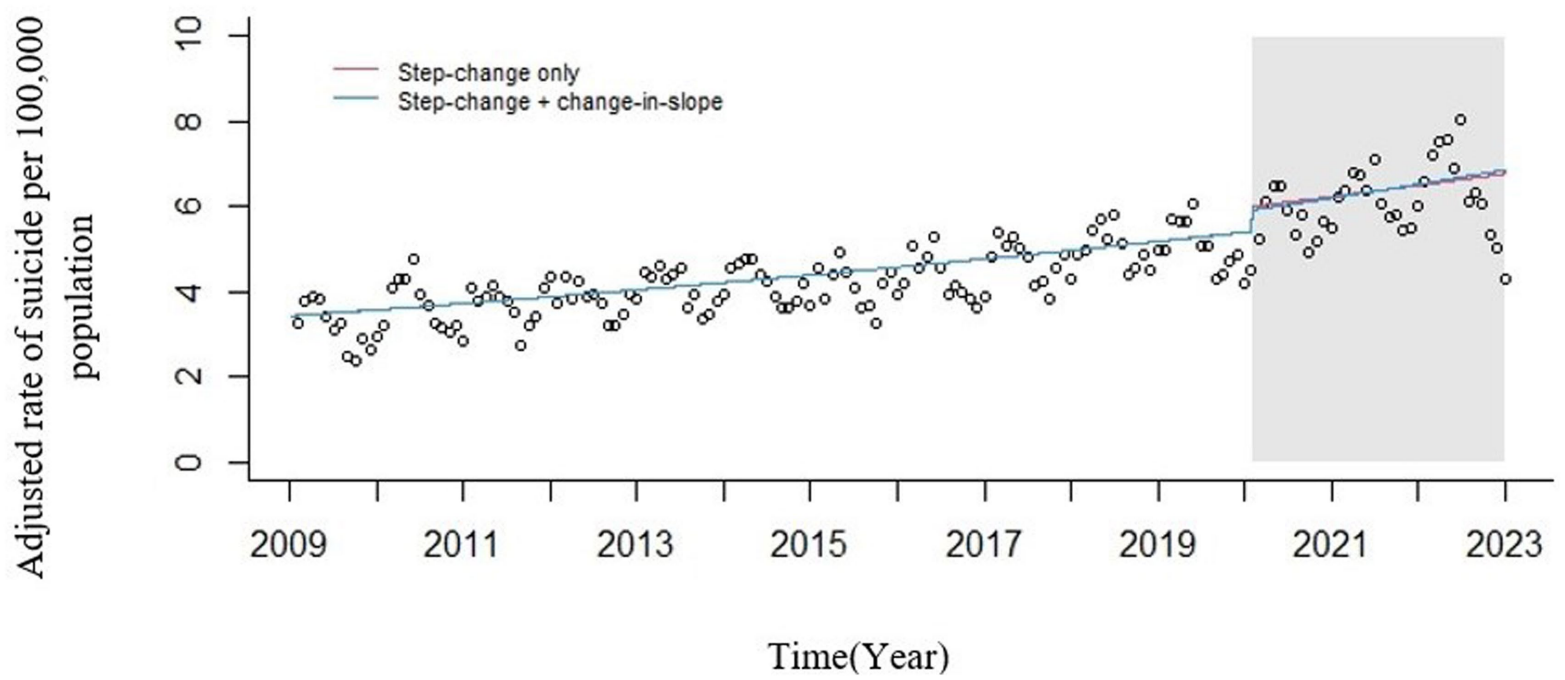
Seasonally adjusted ITS plot of standardized incidence of suicide deaths in IRAN from March 2009 to 2023. Circles = observed rates; red line = modeled rates fitted to the data (step change only); blue line = modeled rates fitted to the data (multiplicative interaction). Gray area = Announcement of mental health orientation following the COVID-19 pandemic by the WHO.

According to Figure 3 and the results of the regression performed, it is observed that the COVID-19 pandemic has only caused an increase in the level of suicide cases (immediate effect) and has not changed the slope of the graph until March 2023.

## Discussion

The main focus of the discussion in this study was to investigate the effect of the COVID-19 pandemic on the trend of suicides leading to death before and after the pandemic, and regarding the reasons for the increase or decrease in suicides according to the variables briefly mentioned in the descriptive results, another article is being reviewed and written by the research team, and the discussion about them was avoided.

COVID-19 in Iran started with two deaths on February 19, 2020, and increased to 145,113 cases by March 20, 2023 (30). At the same time, 18,043 cases of suicide deaths were recorded. In May 2020, when the World Health Organization announced that COVID-19, in addition to being an infectious and transmissible disease, is also a mental health emergency, this date was considered the basis for the start of the intervention in this study. The results showed that after three months from the start of the COVID-19 pandemic in Iran, which was equivalent to six months after the presence of the disease in the world, the incidence of suicide deaths throughout the pandemic until the end of 2023, in addition to the increasing trend, changed in the level of suicide incidence. Therefore, the incidence of suicide from May 2020 to March 2023 was accompanied by a 19% increase in suicide, although in the absence of COVID-19, the rate of suicide had an annual increase of 0.3%. Many studies worldwide have examined the effect of COVID-19 on the incidence of mental health diseases, attempted suicide and suicide deaths, and in each country, depending on different economic, social and cultural conditions, this effect has been different. For example, in Japan, to examine the social-economic recession and social isolation due to the spread of COVID-19 disease, the expected suicide mortality rate in 2020 was estimated based on suicide mortality from 2011 to 2019 using Join point regression analysis, and after comparing with the actual suicide mortality rates in Japan in 2020, it was observed that the actual suicide mortality in 2020 (one year after the COVID-19 pandemic in the world) was significantly higher than the expected mortality among Japanese men and women (31). Japan was one of the countries that had been relatively successful in controlling the COVID-19 pandemic among Asian countries. When we examine the study by Luo and colleagues on five successful countries in controlling COVID-19 in Asia, one of these countries is Singapore, which was able to control this disease well and accurately by tracing the contacts of people suspected of having the disease (32). To examine the suicide statistics in Singapore, the nonprofit suicide prevention center of this country announced that suicide has been the main cause of death for young people aged 10 to 29 in Singapore for the fourth consecutive year since 2019, and the number of 476 reported suicides in 2022 indicates the highest rate since 2000 in this country and needs to be investigated (33). According to the report of the Centers for Disease Control and Prevention of the United States, the age-adjusted overall suicide rate increased from 10.7 deaths per 100,000 standard population in 2001 to its maximum level of 14.2 in 2018 and then decreased to 13.5 in 2020. However, again in 2021, this rate increased by 4 percent to 14.1 (34). Of course, this study does not mention the effect of COVID-19 on the rate of suicide, but this 4% increase in the occurrence of suicide after the COVID-19 pandemic can be considered for further studies. One study that has examined the impact of COVID-19 on work-related suicides is the thesis of Mona Hassan, who graduated with a master’s degree from Washington University in 2023. Her thesis focused on the suicide cases that were related to the workplace during the COVID-19 pandemic using data from the National Violent Death Reporting System (NVDRS) in the United States. She found that COVID-19 potentially highlighted work-related suicides and emphasized the importance of considering demographic factors and conditions in understanding and addressing these incidents (35). As you can see, suicide during the COVID-19 pandemic has been different in different countries, and how many suicides were due to the presence of the COVID-19 pandemic and the specific conditions prevailing in societies during this disease may be more accurately determined in the coming years. However, what has been determined in Iran is that the incidence of suicide in the years after the COVID-19 pandemic has continued to increase with an increasing trend and at a higher level than before the COVID-19 pandemic. It is possible that many factors other than the COVID-19 pandemic influenced the increase in the level of suicide during the post-COVID-19 pandemic period.

## The limitations, weaknesses, and strengths of the present study

Among the important limitations and weaknesses that were evident in this study, the following can be mentioned:

Due to the stigma of suicide in many countries, including Iran, there is still the possibility of the survivors of the deceased hiding the cause of death or not cooperating in completing suicide cases. Considering that this study is an ecological study and in recent years, many social, economic, and political events have occurred in Iran whose effects have not been considered in this study, the results should be interpreted more carefully in order to avoid the ecological fallacy bias. Considering that the number of suicide attempts is always several times more than the number of suicides resulting in death, unfortunately, despite correspondence with the relevant organizations in Iran, there was no access to the statistics of suicide attempts, and therefore it was not possible to compare. It is recommended to review and analyze the data and compare the results with other statistical methods such as data analysis and comparison with other statistical methods such as AutoArima in Iran and compare with Iran’s neighboring countries.

One of the most important strengths of this study is that all suspicious deaths due to suicide were collected and recorded by the Forensic Medicine Organization, so the data in question were among the most reliable sources of suicide information in Iran.

## Conclusion

Suicide rarely occurs as a result of a situation or event. A range of different factors (at individual, relational, societal and environmental levels) can increase the risk. Although this phenomenon is also preventable, it has grown significantly with the gradual progress of societies and the transformation of structures, and despite the attention and efforts of the scientific community, including psychiatrists, sociologists, psychologists and social science counselors, in controlling it, unfortunately, we still witness the occurrence of this bitter and unpleasant phenomenon in many societies, including Iran. In addition to all these cases, unexpected and natural factors that suddenly occur in a society, such as floods, earthquakes, volcanoes, and epidemics such as COVID-19, which caused restrictions in relations and the presence of people in society, can create a favorable environment for people to tend to complete suicide or attempt suicide. Therefore, it is necessary for the economic and health policymakers of the society to take actions to reduce the various effects left over from that crisis during and after any crisis. It is hoped that with regard to the results of this study, which showed an increase in suicides leading to death in the presence of the COVID-19 pandemic and considering the sensitivity and importance of suicide phenomenon in any country, including Iran, the officials and planners of the country who always have the concern of reducing health problems, mortality, mental disorders, and social problems of the people of society can identify provinces with an increasing trend of suicide and provide necessary measures to increase people’s access to mental health services. In order to continue the work, it is suggested to investigate the effect of social events in Iran on the phenomenon of suicide in different periods and compare the results with this study.

## Data Availability

The datasets presented in this article are not readily available because the data was received from the legal medicine of Iran, confidentiality and preservation of the data was one of the conditions of the research. Requests to access the datasets should be directed to amahdavi202034@gmail.com.
